# Pathology and Pathogenesis of SARS-CoV-2 Associated with Fatal Coronavirus Disease, United States

**DOI:** 10.3201/eid2609.202095

**Published:** 2020-09

**Authors:** Roosecelis B. Martines, Jana M. Ritter, Eduard Matkovic, Joy Gary, Brigid C. Bollweg, Hannah Bullock, Cynthia S. Goldsmith, Luciana Silva-Flannery, Josilene N. Seixas, Sarah Reagan-Steiner, Timothy Uyeki, Amy Denison, Julu Bhatnagar, Wun-Ju Shieh, Sherif R. Zaki, COVID-19 Pathology Working Group

**Affiliations:** Centers for Disease Control and Prevention, Atlanta, Georgia, USA (R.B. Martines, J.M. Ritter, E. Matkovic, J. Gary, B.C. Bollweg, C.S. Goldsmith, L. Silva-Flannery, J.N. Seixas, S. Reagan-Steiner, T. Uyeki, A. Denison, J. Bhatnagar, W.-J. Shieh, S.R. Zaki);; Synergy America Inc., Atlanta (H. Bullock).

**Keywords:** SARS-CoV-2, COVID-19, coronavirus, pathology, histopathology, immunohistochemistry, electron microscopy, diffuse alveolar damage, respiratory infections, viruses, 2019 novel coronavirus disease, severe acute respiratory syndrome coronavirus 2, zoonoses, coronavirus disease

## Abstract

An ongoing pandemic of coronavirus disease (COVID-19) is caused by infection with severe acute respiratory syndrome coronavirus 2 (SARS-CoV-2). Characterization of the histopathology and cellular localization of SARS-CoV-2 in the tissues of patients with fatal COVID-19 is critical to further understand its pathogenesis and transmission and for public health prevention measures. We report clinicopathologic, immunohistochemical, and electron microscopic findings in tissues from 8 fatal laboratory-confirmed cases of SARS-CoV-2 infection in the United States. All cases except 1 were in residents of long-term care facilities. In these patients, SARS-CoV-2 infected epithelium of the upper and lower airways with diffuse alveolar damage as the predominant pulmonary pathology. SARS-CoV-2 was detectable by immunohistochemistry and electron microscopy in conducting airways, pneumocytes, alveolar macrophages, and a hilar lymph node but was not identified in other extrapulmonary tissues. Respiratory viral co-infections were identified in 3 cases; 3 cases had evidence of bacterial co-infection.

The ongoing global pandemic of coronavirus disease (COVID-19), caused by severe acute respiratory syndrome coronavirus 2 (SARS-CoV-2), was identified in Wuhan, Hubei Province, China, and has spread rapidly around the world (*1*,*2*). As of May 18, 2020, World Health Organization official data reported 4,628,903 confirmed cases and 312,009 deaths (*2*). On January 20, 2020, the Centers for Disease Control and Prevention (CDC) confirmed a case in the United States; since then, all 50 US states, District of Columbia, Guam, Puerto Rico, Northern Mariana Islands, and US Virgin Islands have confirmed cases of COVID-19 (*2**–**4*). 

Coronaviruses are enveloped, positive-stranded RNA viruses that infect many animals; human-adapted viruses likely are introduced through zoonotic transmission from animal reservoirs (*5*,*6*). Most known human coronaviruses are associated with mild upper respiratory illness. SARS-CoV-2 belongs to the group of betacoronaviruses that includes severe acute respiratory syndrome coronavirus (SARS-CoV) and Middle East respiratory syndrome coronavirus (MERS-CoV), which can infect the lower respiratory tract and cause a severe and fatal respiratory syndrome in humans (*7*). SARS-CoV-2 has >79.6% similarity in genetic sequence to SARS-CoV (*5*).

 SARS-CoV-2 is highly transmissible among humans; fatality rates for COVID-19 vary and are higher among the elderly and persons with underlying conditions or immunosuppression (*8*,*9*). The current knowledge about COVID-19 pathogenesis and pathology in fatalities is based on a small number of described cases and extrapolations from what is known about other similar coronaviruses, such as SARS-CoV and MERS-CoV (*10**–**18*). Pathologic evaluation and determination of virus distribution and cellular localization within tissues is crucial to elucidating the pathogenesis of these fatal infections and can help guide development of therapeutic and preventive countermeasures. We report on the histopathologic features and detection of virus in tissues by immunohistochemistry (IHC) and electron microscopy (EM) from 8 confirmed fatal cases of COVID-19 in the United States.

## Materials and Methods

### Study Patients and Data Collection

As part of the public health response to COVID-19, the CDC Infectious Diseases Pathology Branch (Division of High-Consequence Pathogens and Pathology, National Center for Emerging and Zoonotic Infectious Diseases) was consulted on autopsies of 8 patients with laboratory evidence of SARS-CoV-2 by reverse transcription PCR (RT-PCR) on respiratory swab specimens collected either before or after death. We reviewed available medical records and preliminary autopsy reports for information regarding demographics, symptom history, underlying conditions, infectious disease testing, imaging study findings, treatment and advanced supportive care received, and date of death. This investigation was reviewed in accordance with CDC’s human subjects review procedures and was determined to not meet the definition of research.

### Histopathology and Immunohistochemistry

We performed routine hematoxylin–eosin stains for histopathologic evaluation. We conducted an IHC assay for SARS-CoV-2 using a rabbit polyclonal antibody raised against SARS-CoV nucleocapsid (Novus Biologicals, https://www.novusbio.com) (*19*) at 1:100 dilution and a Mach 4 Universal AP Polymer Kit (Biocare Medical, https://biocare.net) with Permanent Red Chromogen (Cell Marque/Millipore Sigma, https://www.cellmarque.com). We pretreated the slides with heat-induced epitope retrieval with a citrate-based buffer (Biocare Medical). We ran appropriate negative controls in parallel, using normal rabbit serum in place of the primary antibody. We validated cross reactivity of the anti–SARS-CoV antibody with SARS-CoV-2 by testing controls created from SARS-CoV-2–infected Vero cells embedded with normal human tissues; we used this control as the positive control for subsequent IHC assays. The SARS-CoV nucleocapsid antibody did not cross-react with influenza A(H1N1) virus, influenza B virus, respiratory syncytial virus, parainfluenza virus type 3, human coronavirus (HCoV) 229E, or MERS-CoV in PCR-confirmed tissue samples. For cases with bronchopneumonia, we performed IHC testing for bacterial agents using a mouse monoclonal antibody raised against *Streptococcus pneumoniae* but known to also detect other *Streptococcus* spp. and a rabbit polyclonal anti-*Klebsiella pneumoniae* antibody (both from Thermo Fisher, https://www.thermofisher.com) known to also detect other gram-negative bacteria (*Escherichia coli*, *Haemophilus influenzae*, and *Pseudomonas* spp.).

For double-stained assays, we used Envision G/2 Double Stain System, Rabbit/Mouse (DAB Permanent Red) from Agilent Technologies (https://www.agilent.com). We used antibodies against CD163 (Leica Biosystems, https://www.leicabiosystems.com) or surfactant apoprotein A (Dako, https://www.agilent.com), followed by the anti–SARS-CoV nucleocapsid antibody (Novus Biologicals). We performed all assays according to the manufacturer’s guidelines. We used SARS-CoV-2–infected Vero cells as a positive control and used non–COVID-19 cases and normal rabbit serum in place of primary antibody as negative controls.

### Electron Microscopy

We obtained upper airway and lung tissue specimens from formalin-fixed samples, cut them into cubes, rinsed them with 0.1 mmol/L phosphate buffer, postfixed them with 2.5% glutaraldehyde, and rinsed them in phosphate buffer. In addition, we removed tissue samples from areas corresponding to positive SARS-CoV-2 immunostaining from paraffin blocks with a 2-mm punch or from 4-μm sections on glass slides; we deparaffinized the samples in xylene and rehydrated. We processed tissues for transmission EM as described previously (*20*). We immersed the sections embedded in epoxy resin in boiling water, removed them from the slides with a razor blade, and cut out the areas of interest and glued them onto a blank EM block. We stained EM sections with uranyl acetate and lead citrate and examined them on a Thermo Fisher/FEI Tecnai Spirit or Tecnai BioTwin electron microscope.

### RT-PCR

We extracted nucleic acids from formalin-fixed paraffin-embedded (FFPE) tissues and assessed them by a conventional RT-PCR specifically targeting the nucleocapsid gene of SARS-CoV-2 (J. Bhatnagar, unpub. data) and real-time RT-PCR/PCR targeting other respiratory pathogens, including influenza viruses, respiratory syncytial virus, human parainfluenza viruses, and *Streptococcus pneumoniae*, as described previously (*21*,*22*). The SARS-CoV-2 nucleocapsid assay detects SARS-CoV-2 and SARS-CoV but does not amplify MERS-CoV and other common human coronaviruses, including alphacoronavirus (HCoV-NL63) and betacoronavirus (HCoV-HKU1).

## Results

### Clinical Data

Of the 8 case-patients, 7 were residents of a long-term care facility (LTCF) in Washington state (Table 1) (*23*,*24*). Seven (87.5%) were White, non-Hispanic. The median age of the 8 case-patients was 73.5 years; 2 were <65 years of age. The median number of days from illness onset until death was 12.5 (range 6–15). Common signs and symptoms reported included fever (75%), cough (62.5%), and shortness of breath (62.5%). All patients had abnormal findings on chest radiographs. Case-patients were all hospitalized for a median of 3.5 days. Six (75%) patients received mechanical ventilation; 2 received comfort care. Underlying medical conditions were identified in all case-patients; hypertension (75%), chronic kidney disease (75%), cardiovascular disease (75%), obesity (62.5%), and diabetes (50%) were the most frequent conditions reported.

**Table 1:** Selected demographic and clinical characteristics of 8 case-patients with fatal severe acute respiratory syndrome coronavirus 2 infection

Characteristic	No. (%)
Sex	
M	4 (50)
F	4 (50)
Age group, y	
<65	2 (25)
>65	6 (25)
Race/ethnicity	
White, non-Hispanic	7 (87.5)
Hispanic	1 (12.5)
Clinical symptoms	
Fever	6 (75)
Cough	5 (62.5)
Shortness of breath	5 (62.5)
Malaise	2 (25)
Myalgias	1 (12.5)
Diarrhea	1 (12.5)
Underlying health conditions	
>1 condition	8 (100)
Hypertension	6 (75)
Chronic kidney disease*	6 (75)
Cardiovascular disease†	6 (75)
Obesity‡	5 (62.5)
Diabetes mellitus	4 (50)
Chronic lung disease§	2 (25)
Immunocompromised condition¶	3 (37.5)
Neurologic disorders	1 (12.5)
Other chronic diseases#	6 (75)
Radiographic findings	
Bilateral interstitial infiltrate or opacities	8 (100)
Pleural effusion	2 (25)
Clinical course	
Median days from illness onset to death (range)	12.5 (6–15)
Median days of hospitalization (range)	3.5 (1–8)
Mechanical ventilation**	6 (75)

*Chronic kidney disease requiring treatment with hemodialysis was reported in 2 case-patients. †Number of cases includes patients with history of coronary artery disease, congestive heart failure, valvular heart disease, and/or cerebrovascular accident. ‡Obesity includes reported BMI >30 or mention of obesity or morbid obesity in the submitted medical records. Two patients had morbid obesity listed in the submitted medical records. §Chronic obstructive pulmonary disease was the only reported underlying condition with chronic lung disease. ¶This includes persons immunosuppressed by solid organ transplant, corticosteroid therapy, or immunosuppressive medication. #Includes any of the following: hyperlipidemia, thyroid disease, rheumatologic disorder, and obstructive sleep apnea. **Two patients were transitioned to comfort care and were not intubated.

### Histopathology, Immunohistochemistry, and Electron Microscopy

**Figure 1:**
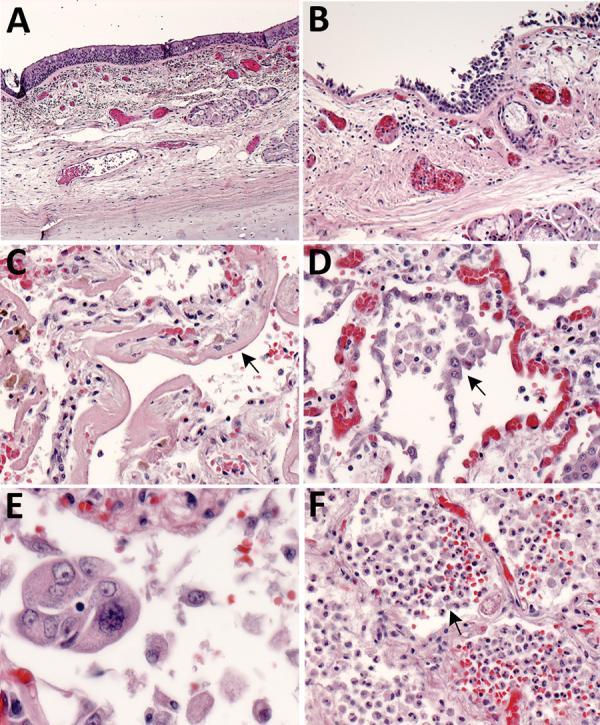
Pulmonary histopathology in fatal coronavirus disease cases caused by severe acute respiratory syndrome coronavirus 2 infection. A) Patient no. 5: tracheitis characterized by moderate mononuclear inflammation within the submucosa (original magnification ×10). B) Patient no. 3: extensive denudation of tracheal epithelium; submucosal congestion, mild edema, and mononuclear inflammation (original magnification ×10). C) Patient no. 4: exudative phase of diffuse alveolar damage characterized by abundant hyaline membranes lining alveolar spaces (arrow) (original magnification ×20). D) Patient no. 8: proliferative phase of diffuse alveolar damage characterized by proliferation of type II pneumocytes (arrow) (original magnification ×20). E) Patient no. 1: atypical pneumocytes with enlarged and multiple nuclei, and expanded cytoplasm in a case with proliferative DAD (original magnification ×40). F) Patient no. 7: bronchopneumonia with filling of alveolar spaces by neutrophils and patchy hemorrhage (arrow) (original magnification ×10).

**Figure 2:**
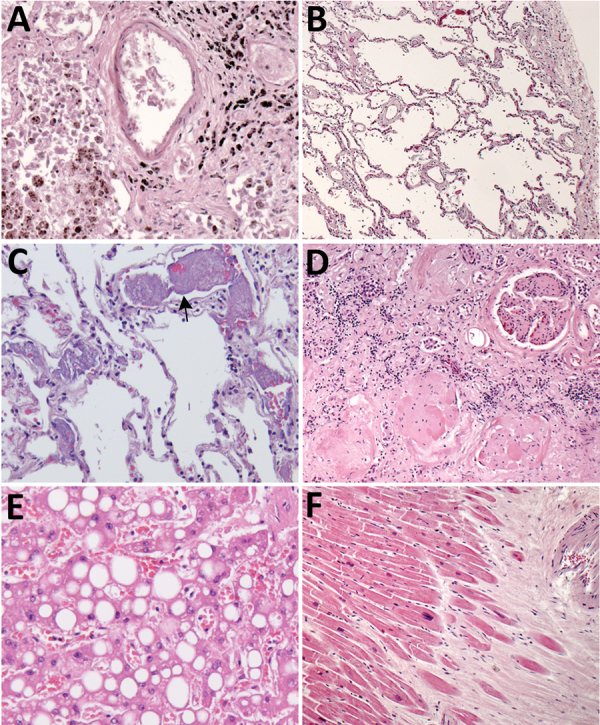
Histopathologic findings associated with underlying conditions in fatal coronavirus disease. A) Patient no. 2: lung, hemosiderin-laden macrophages (brown pigment, bottom left), and anthracosis (black pigment, top right) in a patient with congestive heart failure (original magnification ×20). B) Patient no. 3: lung, emphysema in a patient with chronic obstructive pulmonary disease (original magnification ×5). C) Patient no. 7: lung, pulmonary microthrombosis (arrow) (original magnification ×20). D) Patient no. 2: kidney, extensive glomerulosclerosis in a patient with renal disease (original magnification ×10). E) Patient no. 3: liver, steatosis in a patient with morbid obesity (original magnification ×20). F) Patient no. 2: heart, myocardial fibrosis and mild cardiomyocyte hypertrophy in a patient with cardiomegaly (original magnification ×5).

Histopathologic findings and results of testing performed on FFPE tissues showed mild to moderate tracheobronchitis was consistently present and characterized by mononuclear inflammation, with epithelial denudation and submucosal congestion (Table 2; Figure 1, panels A, B). The predominant lung pathology was diffuse alveolar damage (DAD); acute phases, organizing phases, or both were present in 7 (87.5%) of 8 patients. Desquamation of pneumocytes and the presence of hyaline membranes, alveolar edema and fibrin deposits, type II pneumocyte hyperplasia, and alveolar infiltrates, including increased alveolar macrophages, were seen (Figure 1, panels C, D). Squamous metaplasia and atypical pneumocytes were present in 3 case-patients, and rare multinucleated cells were present in 1 case-patient (patient no. 1) (Figure 1, panel E); no definitive viral inclusions were seen. One case-patient without DAD (patient no. 7) had diffuse bronchopneumonia with filling of alveolar spaces by mixed inflammation with abundant neutrophils (Figure 1, panel F). Three additional case-patients had focal bronchopneumonia and increased pulmonary intravascular leukocytes. Hemosiderin-laden macrophages (4/8), hemorrhage (4/8), mucus aspiration (3/8), emphysema (2/8), and microthrombi (1/8) were seen (Figure 2). Anthracosis, common in elderly persons as a result of chronic carbon accumulation, was present in the lungs and pulmonary hilar lymph nodes in all cases. In 6 cases, lymph nodes also showed sinus histiocytosis and hemophagocytosis in subcapsular sinuses. Notable pathologic findings in extrapulmonary tissues included evidence of chronic renal disease (5/8), acute renal tubular injury (3/8), hepatic steatosis (4/8) and cirrhosis (1/8), and focal myocardial fibrosis (3/8) (Figure 2). No myocarditis or myocardial necrosis and no notable histopathologic changes in the intestine were seen in any case. Brain tissues were not available for histopathologic evaluation or testing.

**Table 2:** Histopathologic features and severe acute respiratory syndrome coronavirus 2 detection in respiratory tissues from 8 coronavirus disease fatalities*

Patient no.	1	2	3	4	5	6	7	8
Minimum symptom duration, d	13	10	5	13	16	11	12	7
Tracheobronchitis	++	+	+	+	+	+	++	+
DAD, acute	+	+	++	++	–	–	–	+++
DAD, organizing	+++	+	++	++	+	+	–	+++
Squamous metaplasia	+	–	–	+	–	–	–	+
Atypical pneumocytes	+++	–	–	+	–	–	–	+
Interstitial pneumonitis	+	–	–	+	–	++	–	+
Bronchopneumonia	–	–	+	+	+	–	+++	–
Intravascular leukocytosis	–	–	+	+	+	–	–	–
Anthracosis	+	+	+	++	++	++	+	++
Other lung pathology	–	HLM	Hemorrhage, emphysema, MA	HLM, MA	HLM, MA	Hemorrhage, corpora amylacea	Hemorrhage, HLM, microthrombi	Hemorrhage, emphysema
SARS-CoV-2 IHC, upper airway	–	–	–	–	**	**	*	*
SARS-CoV-2 IHC, lung	–	+	+++	+++	+++	+	++	++
Electron microscopy, viral particles	NA	NA	+	+	+	+	NA	NA
Other agents detected	Influenza B	–	–	*Streptococcus* spp.	PI-3	*Streptococcus* spp.	PI-3	*Streptococcus *spp.

*DAD, diffuse alveolar damage; HLM, hemosiderin-laden macrophages; IHC, immunohistochemistry; MA, mucus aspiration; NA, not available; PI, parainfluenza; – , not present or negative; +, mild; ++, moderate; +++, extensive.

**Figure 3:**
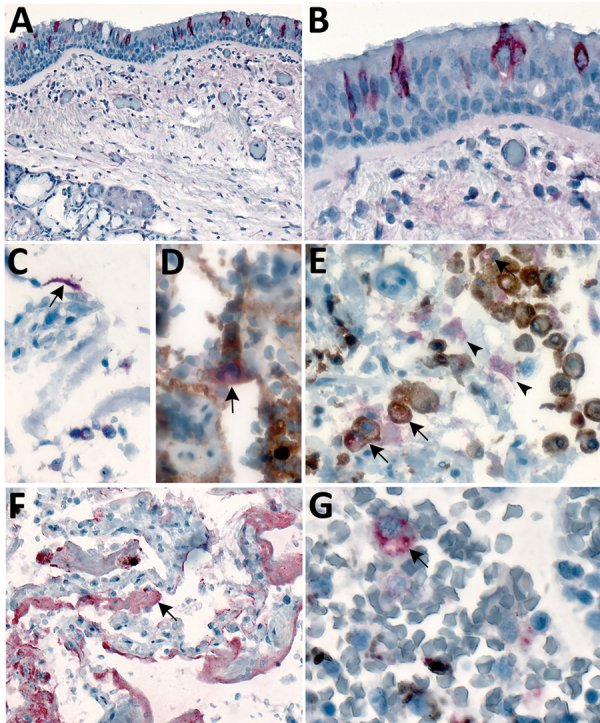
Immunostaining of severe acute respiratory syndrome coronavirus 2 in pulmonary tissues from fatal coronavirus disease cases. A) Patient no. 5: scattered immunostaining of tracheal epithelial cells (original magnification ×40). B) Patient no. 5: higher magnification shows immunostaining of ciliated cells (original magnification ×63). C) Patient no. 8: immunostaining of desquamated type I pneumocyte in an alveolar lumen (original magnification ×63). D) Patient no. 4: colocalization of SARS-CoV-2 viral antigen (red) with type II pneumocyte stained by surfactant (brown; arrow) (original magnification ×63). E) Patient no. 4: colocalization of SARS-CoV-2 viral antigen (red) with macrophages stained by CD163 (brown; arrows); virus immunostaining within type II pneumocytes is also seen (arrowheads) (original magnification ×40). F) Patient no. 4: extensive immunostaining of hyaline membranes in a region of exudative DAD (original magnification ×20). G) Patient no. 3: scattered immunostaining within macrophage in hilar lymph node; anthracosis is also present (original magnification ×63).

We detected SARS-CoV-2 by IHC in the upper airways in 4/8 (50%) case-patients and in the lungs in 7/8 (92%) case-patients. We observed immunostaining of viral antigens in upper airway and bronchiolar epithelium, submucosal gland epithelium, and in type I and type II pneumocytes, alveolar macrophages, and hyaline membranes in the lung (Figure 3, panels A–C, F). Upper airways and lung tissues from all 8 case-patients were positive by SARS-CoV-2 RT-PCR. Double staining with surfactant showed colocalization of SARS-CoV-2 antigen with type II pneumocytes (Figure 3, panel D); double staining with CD-163 showed viral antigen colocalization with macrophages (Figure 3, panel E). We also found viral immunostaining in scattered macrophages in the hilar lymph node from 1 severely immunosuppressed patient with a history of solid-organ transplant (Figure 3, panel G). We did not detect SARS-CoV-2 by IHC in heart, liver, kidney, spleen, or intestine from any patient.

Six (75%) of 8 case-patients had either viral or bacterial co-infections, but not both, identified by IHC, PCR, or both in addition to SARS-CoV-2. Respiratory viral PCR testing detected parainfluenza virus type 3 coinfection in upper airway and lung tissue in 2/8 (25%) case-patients and influenza B virus coinfection in upper airway in 1 case-patient. Three (75%) of the 4 case-patients with SARS-CoV-2 and bronchopneumonia had immunostaining for *Streptococcus* spp. Two of these patients had nonpneumococcal *Streptococcus* spp*.* confirmed by PCR testing.

**Figure 4:**
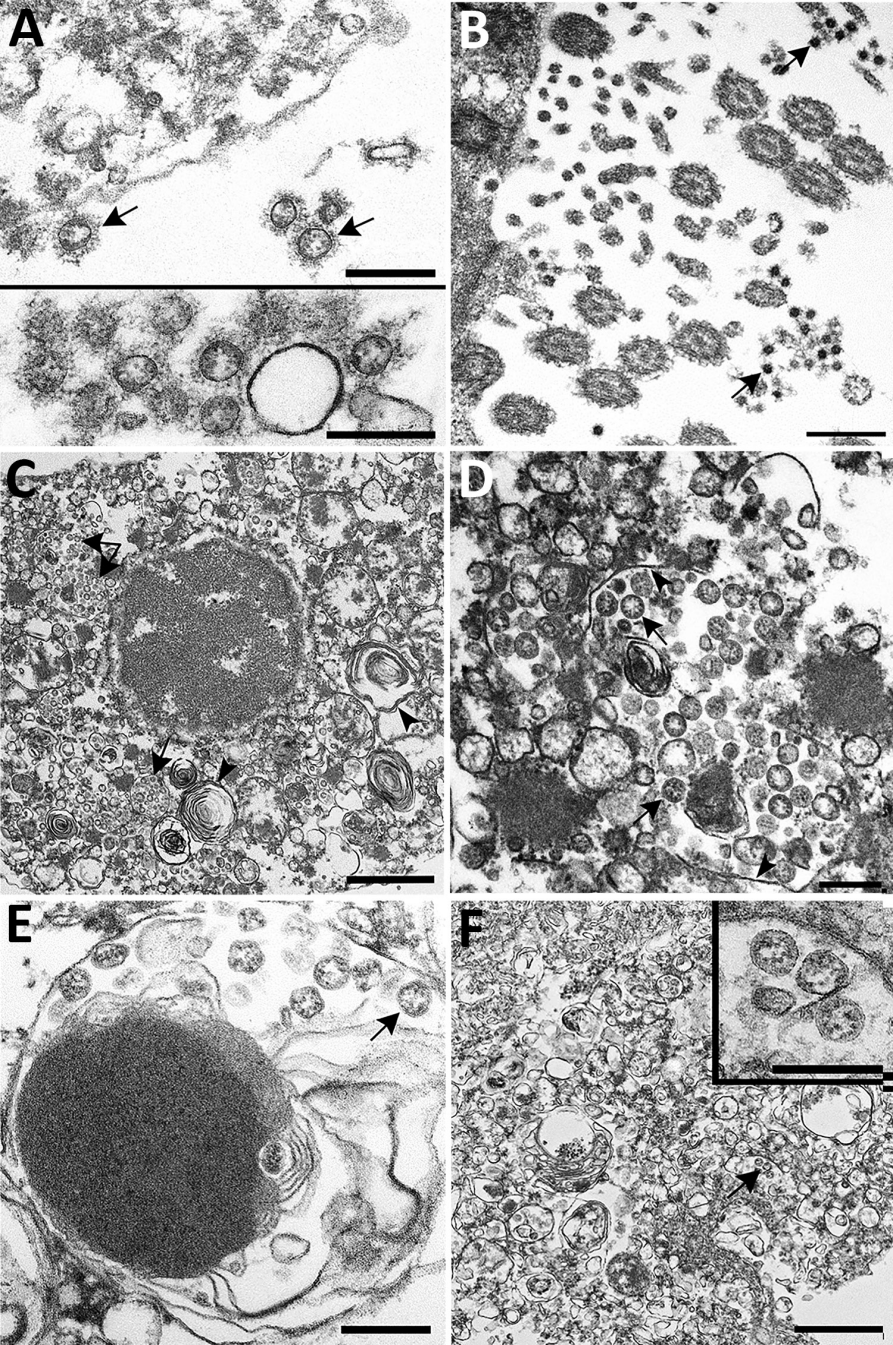
Ultrastructural features of severe acute respiratory syndrome coronavirus 2 lung infection in fatal coronavirus disease. A) Top: alveolar space containing extracellular virions (arrows) with prominent surface projections. Bottom: cluster of virions in the alveolar space. Scale bars indicate 200 nm. B) Extracellular virions (arrow) associated with ciliated cells of the upper airway. Scale bar indicates 200 nm. C) Membrane-bound vacuoles (arrows) containing viral particles within the cytoplasm of an infected type II pneumocyte; surfactant (lamellated material) indicted by arrowheads. Scale bar indicates 1 μm. D) Membrane-bound vacuole (double-headed arrow in panel C) containing virus particles (arrows) with the characteristic black dots that are cross-sections through the viral nucleocapsid. Arrowheads indicate vacuolar membrane. Scale bar indicates 200 nm. E) Viral particles (arrow) within a phagosome of an alveolar macrophage. Scale bar: 200 nm. F) Viral particles within a portion of a hyaline membrane. Scale bar indicates 800 nm. Inset: Higher magnification of virus particles indicated by arrow; scale bar indicates 200 nm.

**Figure 5:**
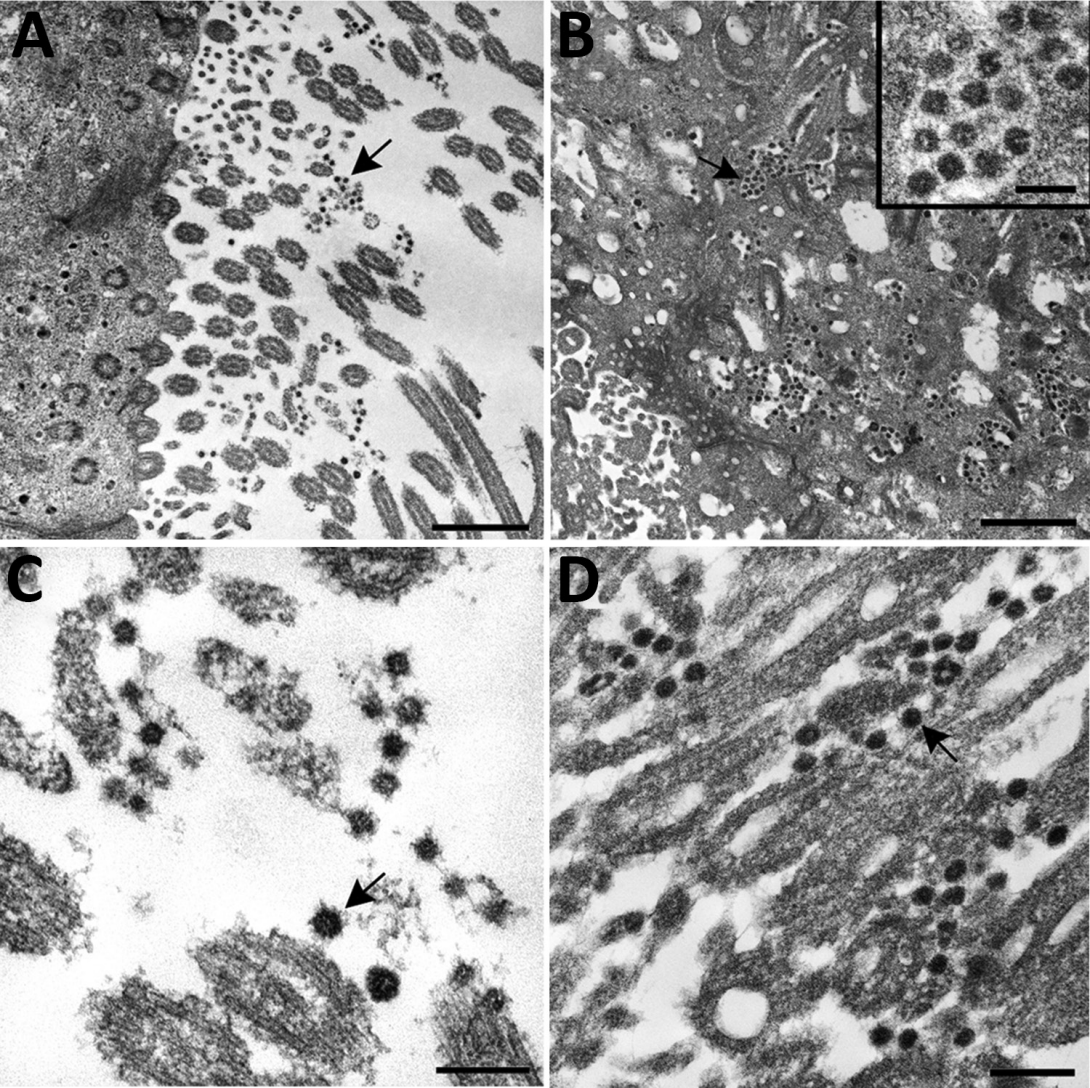
Ultrastructural features of severe acute respiratory syndrome coronavirus 2 infection within the upper airway of a fatal coronavirus disease case from formalin-fixed paraffin-embedded (FFPE) tissue. Viral particles associated with the cilia of ciliated cells (A, C, and D) and the cytoplasm of respiratory epithelial cells (B) in the upper airway are indicted by arrows. Images in panels A and C were obtained from FFPE tissue removed from a paraffin block using a 2-mm biopsy punch. Images in panels B and D were collected from a 3 μm section of FFPE tissue affixed to a glass slide. Viral particles visualized in FFPE samples were smaller than those observed from fresh tissue; extracellular viral particles in fresh tissue samples were 105 nm in diameter and those from FFPE tissues were 75 nm in diameter. Scale bars indicate 1 μm (panel A), 800 nm (panel B), and 200 nm (panels C and D).

EM examination of respiratory tissues showed virions with prominent surface projections (spikes) characteristic of the family *Coronaviridae*. In the lung, extracellular virions free in the alveolar space were, on average, 105 nm in diameter, including surface projections (Figure 4, panel A). In upper airways, virions were seen extracellularly among the cilia and within the cytoplasm of respiratory epithelial cells (Figure 4, panel B; Figure 5). Intracellular virions in type II pneumocytes (Figure 4, panels C, D) and in cytoplasmic vesicles or phagosomes of alveolar macrophages (Figure 4, panel E) were, on average, 75 nm in diameter and lacked prominent spikes. Viral particles were also found associated with fibrin or hyaline membranes within alveolar spaces (Figure 4, panel F).

## Discussion

The clinical distinction between SARS-CoV-2 and other respiratory viral infections is difficult because there are overlapping clinical features characterized by febrile illness with cough that lasts for several days before progressing to acute pneumonia. In addition, persons with SARS-CoV-2 and other respiratory viral infections may have atypical or minimal symptoms (*25**–**27*). Besides respiratory failure, particularly in patients with severe disease, fatigue, myalgia or arthralgia, chills, hepatic and renal dysfunction, lymphocytopenia, leukopenia, thrombocytopenia, and elevated inflammatory biomarkers have been described (*5*,*28*,*29*).

Histopathologic lesions attributed directly to the virus in these cases were limited to respiratory tissues; the predominant finding was DAD, with various levels of progression and severity. We saw no clear correlation of the pathologic phase of DAD to known symptom duration, which could be the result of underrecognition of early symptoms in elderly residents of LTCFs and underestimation of illness duration. Together, the histopathologic, IHC, and EM findings in this report provide insight into SARS-CoV-2 pathogenesis. IHC testing, including double staining with surfactant, and EM confirmed viral tropism for pulmonary II pneumocytes. The ultrastructural observations are consistent with previous reports of SARS-CoV infection, with the exception that neither double-membrane vesicles nor nucleocapsid inclusions were detected (*15*,*20*). Viral antigen was also seen in respiratory epithelium of conducting airways (trachea, bronchi, and bronchioles) and occasionally in alveolar macrophages; infection of these cell types may be key in viral replication and trafficking. The respiratory epithelium is one of the first cell types encountered by inhaled virus; SARS-CoV-2 antigens were detected by IHC in ciliated epithelial cells from 50% of these case-patients and up to 16 days after known symptom onset. Ultrastructural analysis showed numerous extracellular viral particles along the ciliated surface and within ciliated columnar epithelial cells. These findings corroborate reports of high viral loads in the upper respiratory tract and support the potential for persons infected with SARS-CoV-2 to readily transmit the virus, with prolonged and continued viral shedding in severe cases (*30*,*31*).

Overall pathologic features in these 8 COVID-19 deaths were similar to those seen in SARS-CoV and MERS-CoV infections, and in available COVID-19 reports (*10**–**15*,*18*,*20*,*32*). However, the amount of viral antigen detected by IHC in lung tissue from these cases is more than what we have seen in SARS and MERS (*16*,*18*) cases submitted to our laboratory, and its extensive detection in epithelial cells of the upper respiratory tract is unique among these highly pathogenic coronaviruses (*33*). In addition to direct viral effects on tissues, the immune response to viral infection likely plays a major role in determining clinical outcome, and acute decline in COVID-19 patients has been linked to an immune-mediated cytokine storm (*34*). Preliminary evaluation of immune cell populations in the respiratory tissues from these 8 cases revealed abundant T lymphocytes in the upper airways and lung parenchyma, with B lymphocytes in smaller numbers and predominating in areas of lymphoid aggregates (data not shown). Further investigation into the roles of these cell populations in COVID-19 is needed. In 6 of the 8 case-patients, sinus histiocytosis and hemophagocytosis were seen in hilar lymph nodes. However, SARS-CoV-2 antigens were detected by IHC in hilar lymph node macrophages from only 1 immunosuppressed patient. Lymph nodes are key sites for immune recognition and elimination of respiratory pathogens. Elucidating the immune response to, and the effects of immunosuppression on, SARS-CoV-2 infection is therefore of fundamental importance.

SARS-CoV-2 uses the angiotensin-converting enzyme 2 (ACE2) receptor to facilitate viral entry into target cells. ACE2 is expressed in multiple tissues throughout the body, including type II pneumocytes, myocardial cells, cholangiocytes, enterocytes, and oral mucosal epithelium (*5*,*35*,*36*). However, among these patients, SARS-CoV-2 antigens were not detected in extrapulmonary tissues besides hilar lymph node, and pathologic findings in other tissues were attributable to other underlying concurrent conditions. Some of the underlying conditions in these case-patients (e.g., hypertension, COPD) are associated with upregulation of ACE2 receptors; possible correlation of these conditions with COVID-19 severity warrants further exploration (*5*,*28*,*37*). COVID-19 cardiomyopathy and acute cardiac death during clinical resolution of pulmonary disease have been described (*24*,*38*). However, we did not observe any evidence of myocarditis or myocardial necrosis in the tissues of the 8 case-patients we examined. Reports have been made of coagulation abnormalities and pulmonary vascular perfusion issues without DAD in some COVID-19 patients (*39*,*40*), and we saw microthrombi in the lung from 1 case-patient who lacked DAD but had severe bacterial bronchopneumonia. These various and potentially severe cardiovascular complications of COVID-19 warrant further investigation into the specific mechanisms of SARS-CoV-2–induced cardiovascular injury, homeostatic derangement, or both. 

Clinical studies have reported elevated liver enzymes in patients with COVID-19 (*28*,*29*). The lack of viral detection by IHC in the liver in this investigation suggests that for these case-patients, abnormal biomarkers of hepatic injury may not be the result of direct viral infection of hepatocytes. Gastrointestinal symptoms are not typically a prominent feature of COVID-19 but have been reported, and SARS-CoV-2 has been detected in fecal samples (*41**–**43*). However, no histopathologic findings or SARS-CoV-2 antigens were detected in gastrointestinal tissues, and diarrhea was reported for only 1 of these case-patients.

We identified viral co-infections in upper respiratory tract tissues from 3 case-patients, including 2 with parainfluenza virus 3 and 1 with influenza B virus, but the contribution of these co-infections to pulmonary disease and fatal outcomes is unknown. Although we identified streptococcal lower respiratory infections in 3 case-patients, none were caused by *Streptococcus pneumoniae*, and there was no strict correlation of these infections with mechanical ventilation among these case-patients. Because 7 of 8 case-patients discussed in this report were residents of a LTCF, their exposures and risks for viral and bacterial co-infections may be different from those for other patients. Few community-acquired bacterial infections have been reported in critically ill patients with COVID-19, but co-infections are not frequently reported with SARS and MERS (*17*,*32*,*44*). Co-infections may play a key role in increasing susceptibility to, and illness from, SARS-CoV-2 infection in a LTCF setting. Further investigation into this association, and characterization of the etiologic agents most commonly involved, is warranted and may contribute to improved overall management of COVID-19 disease.

This report describes the specific cellular and extracellular localization of SARS-CoV-2 in respiratory tissues, without any IHC evidence of the virus in other tissues. Although detection of SARS-CoV-2 RNA in blood or serum has been reported (*34*,*41*), we did not find evidence of systemic virus dissemination in these case-patients. Our findings highlight the importance of underlying conditions and pulmonary co-infections in COVID-19; these factors may potentially delay or confound diagnosis and contribute to adverse outcomes. 

A limitation of this study is that 7 of 8 cases were from a single skilled nursing facility; findings may therefore not be representative of community-acquired SARS-CoV-2 infections. However, nosocomial transmission of viruses often parallels community outbreaks, and understanding disease transmission in healthcare settings is crucial (*25*,*45*). None of these case-patients had diagnoses of acute cardiac injury, myocarditis, or cardiomyopathy, so understanding the pathogenesis of cardiac injury with SARS-CoV-2 infection requires additional investigations in fatal cases with evidence of cardiac injury.

No clinical or histopathologic features are specific to SARS-CoV-2 infection. Demonstrating SARS-CoV-2 directly in lung tissue, when taken in context with any other pathology present, is critical to assessing its contribution to mortality. Herein, we establish the utility of IHC as a diagnostic modality for SARS-CoV-2 in FFPE tissues by localizing viral antigens in respiratory tissues from RT-PCR confirmed cases. This diagnostic method is particularly valuable for FFPE specimens from cases in which antemortem or postmortem respiratory swab testing for SARS-CoV-2 was not performed. We also demonstrate virus identification in tissues by EM using various tissue sources (formalin-fixed wet tissue, FFPE blocks, and stained slides). Identification of SARS-CoV-2 cellular tropisms in the respiratory tract represents a crucial step forward in understanding the pathogenesis of SARS-CoV-2 infection and provides some insights relevant to the development of targeted therapeutic and preventive measures to combat COVID-19.
